# Nasal Discharges—I

**Published:** 1909-05-29

**Authors:** 


### Diseases of Children.

### NASAL DISCHARGES—I.

Seeing that the main symptom of affections of the nose and naso-pharynx is a discharge from the anterior nares, it will be useful to review the different conditions which give rise to this symptom. It is one of very common occurrence and does not receive the attention it deserves. Often it is due to an inflammatory affection, which extends backwards, and is liable to set up otitis media through infection of the middle ear by way of the Eustachian tube.

1. *Acute catarrhal rhinitis*, acute coryza, or simple nasal catarrh. This is especially common in new-born infants and during the first three years of life. Its importance is particularly great at this time, for blockage of the nose leads to mouth-breathing, inability to suck, refusal of food, wasting, fatigue, dyspnœa, insomnia, and fever. It is commonly the result of microbial infection, and has been ascribed to the influenza bacillus of Pfeiffer, the bacillus coryzæ segmentosus (Cautley) or bacillus septus, the micrococcus catarrhalis (Kirchner), Hoffmann's bacillus, etc. Probably numerous organisms belonging to the influenza group may give rise to it. Or it may indicate the onset of measles or true influenza. Occasionally one comes across cases of vaso-motor origin, in which profuse watery discharge and much sneezing last for a few hours up to a day or so. More commonly acute coryza runs its course in about one week. Of the predisposing causes we may mention indoor life, heated rooms, closed bedroom windows, excess of clothing, lack of exercise, malnutrition, rickets, and adenoids. Possibly artificial feeding, by lack of suckling, is a concomitant factor by leading to mal-development of the nasal chambers. More probably it acts through imperfect nutrition, because of a deficiency of proteid and fat, and an excess of starchy food in the diet. Congenitally syphilitic babies are also more liable to the affection. The true exciting cause is, without doubt, some organism, perhaps normally an inhabitant of the nose or naso-pharynx, but whose virulence is exaggerated or relatively greater, because of lowered local or general vitality, from chill, wet, cold winds, sweating, etc. Even a strong healthy child, brought up on approved hygienic principles and without a trace of adenoids, may develop attacks of this nature through being out on a cold and windy day and inhaling germ-laden dust. The finely powdered dust of wood pavements seems especially irritating to the mucous membranes. Earache and deafness are frequent symptoms. Extension also takes place to the eyes and lachrymal ducts, and occasionally to the various fossæ. Alimentary disturbance and pulmonary affections are common complications. The obstructive effects are the most serious, for in a weakly babe they may result in atelectasis or even in sudden death.

Active treatment of acute coryza in infants is much more necessary than in later life. Keep the child in bed, in a warm room, at 60° F. to 70° F., and have the air moistened by means of a bronchitis kettle. Place a pledget of absorbent cotton-wool, soaked in 0.001 per cent. adrenalin solution, in each nostril alternately for two or three minutes. The effects last for three or four hours. It may be repeated as often as this, or, in very bad cases of congestion and obstruction, before each feed. A little borated vaseline introduced into the nostrils will clear away mucus by causing sneezing. Give frequent inhalations of ol. pini sylvestris or turpentine dropped on lint. Apply to the edges of the nose and the upper lip, in order to prevent excoriation, cold cream or an ointment of boric acid grs. 20, salicylic acid grs. 3, vaseline one half ounce. If crusts form, anoint with weak white precipitate ointment. Other applications, in place of the adrenalin solution, are a solution of menthol, 1 to 2 per cent. in olive oil; chloral hydrate, grs. 5 to 10 in half an ounce of castor oil, menthol gr. 1, camphor gr. 1, liquid albolene 1 oz.; a 1 to 2 per cent. watery solution of cocaine. Occasionally it is even necessary to insert a small rubber catheter in each nostril to keep the passage open. In later stages, when the discharge has become chronic and purulent, perhaps blood-stained, apply the adrenalin pledgets as before, and drop into each nostril one drop of a 0.5 to 1.0 per cent. solution of silver nitrate. Sometimes better results are obtained by cleaning away the mucus with an alkaline lotion and painting the mucous membrane with silver nitrate solution, which may be as strong as 2 per cent. Insufflations, douches and syringing should be avoided in the treatment of the disease in infancy.

Internally, small doses of aspirin or salicin are most satisfactory. Belladonna or its alkaloid is sometimes given with a view to reducing the secretion. Mild diaphoretics relieve both the fever and the local congestion. The bowels must be opened by grey powder or calomel. The diet should be light and digestible. It may be requisite to feed a debilitated infant by the stomach tube, and to give alcohol and strychnia freely. Temporary starvation is valuable for strong children. Older children are treated on similar lines, and with mustard foot-baths, hot diaphoretic drinks, and Dover's powder at the onset. Aspirin, salicylates, sodium benzoate in one-drachm doses, and alkalies should be given. Inhalations of turpentine, ol. pini sylvestris, ol. eucalypti, formalin, formaldehyde and menthol, or menthol and camphor with eucalyptus oil, are all recommended. Or a 10 to 20 per cent. borated vaseline is snuffed up into the nose, which is blown at the end of ten minutes. In blowing the nose, under all circumstances, only one nostril should be closed at a time. Protargol, 10 per cent., may be sprayed or painted on the nasal mucosa once or twice in the early stages; or a paint of cocaine, 2 per cent., in glycerin or liquid vaseline. Various powders are used as snuffs for adults, but are rarely advisable for children. A piece of ointment, the size of a pea, made of one or more of the powders of salol 5 gr., boric acid 1 dr., menthol 5 gr., resorcin 10 gr., camphor 1 dr. to the ounce of vaseline, can be inserted into the nostrils twice a day.

*(To be continued.)*

